# Dietary patterns interfere with gut microbiota to combat obesity

**DOI:** 10.3389/fnut.2024.1387394

**Published:** 2024-06-17

**Authors:** Xiaofan Lou, Pusen Li, Xiaoyan Luo, Zhu Lei, Xudong Liu, Yang Liu, Lulu Gao, Weiwei Xu, Xiaomeng Liu

**Affiliations:** Nutrition and Food Hygiene Laboratory, School of Public Health, Xinxiang Medical College, Xinxiang, China

**Keywords:** dietary patterns, obesity, gut microbiota, metabolism, intervention

## Abstract

Obesity and obesity-related metabolic disorders are global epidemics that occur when there is chronic energy intake exceeding energy expenditure. Growing evidence suggests that healthy dietary patterns not only decrease the risk of obesity but also influence the composition and function of the gut microbiota. Numerous studies manifest that the development of obesity is associated with gut microbiota. One promising supplementation strategy is modulating gut microbiota composition by dietary patterns to combat obesity. In this review, we discuss the changes of gut microbiota in obesity and obesity-related metabolic disorders, with a particular emphasis on the impact of dietary components on gut microbiota and how common food patterns can intervene in gut microbiota to prevent obesity. While there is promise in intervening with the gut microbiota to combat obesity through the regulation of dietary patterns, numerous key questions remain unanswered. In this review, we critically review the associations between dietary patterns, gut microbes, and obesity, aiming to contribute to the further development and application of dietary patterns against obesity in humans.

## 1 Introduction

Since 1975, the global number of people who are obese has nearly tripled (1). In 2016, more than 1.9 billion adults above the age of 18 were overweight, with more than 650 million being obese, and over 340 million children and adolescents aged 5–19 are overweight or obese (2). Obesity is increasingly posing a significant threat to global health, increasing the risk of developing non-communicable diseases such as type 2 diabetes mellitus (T2DM), cardiovascular disease, some cancers, and other illnesses (3). Diet plays a crucial role in the development of these diseases. For instance, a Western-type diet, rich in fat and protein but lacking in fiber, vitamins, and minerals (4), can cause the body to take in too much energy. Chronic high-calorie intake combined with a sedentary lifestyle promotes the development of obesity (5).

Dietary patterns refer to the quantity, proportion, type, or combination of different foods in a diet and the frequency with which they are usually consumed (6). Many studies have shown that dietary patterns are closely related to physical health, and many patterns have been developed to treat and prevent obesity and related metabolic diseases. For instance, the Mediterranean diet (MD) can prevent obesity, cardiovascular disease and type 2 diabetes (7). Ketogenic diets (KDs) can induce fat loss (8). Dietary approaches to stop hypertension can prevent and control hypertension (9). Calorie restricted (CR) diet delays aging and reduces the incidence of age-related diseases (10). Intermittent fasting can promote metabolic health (11). The proper use of these dietary patterns and controlled calorie intake can lead to healthy growth and development while reducing the risk of preventable diseases (12).

**Figure 1:**
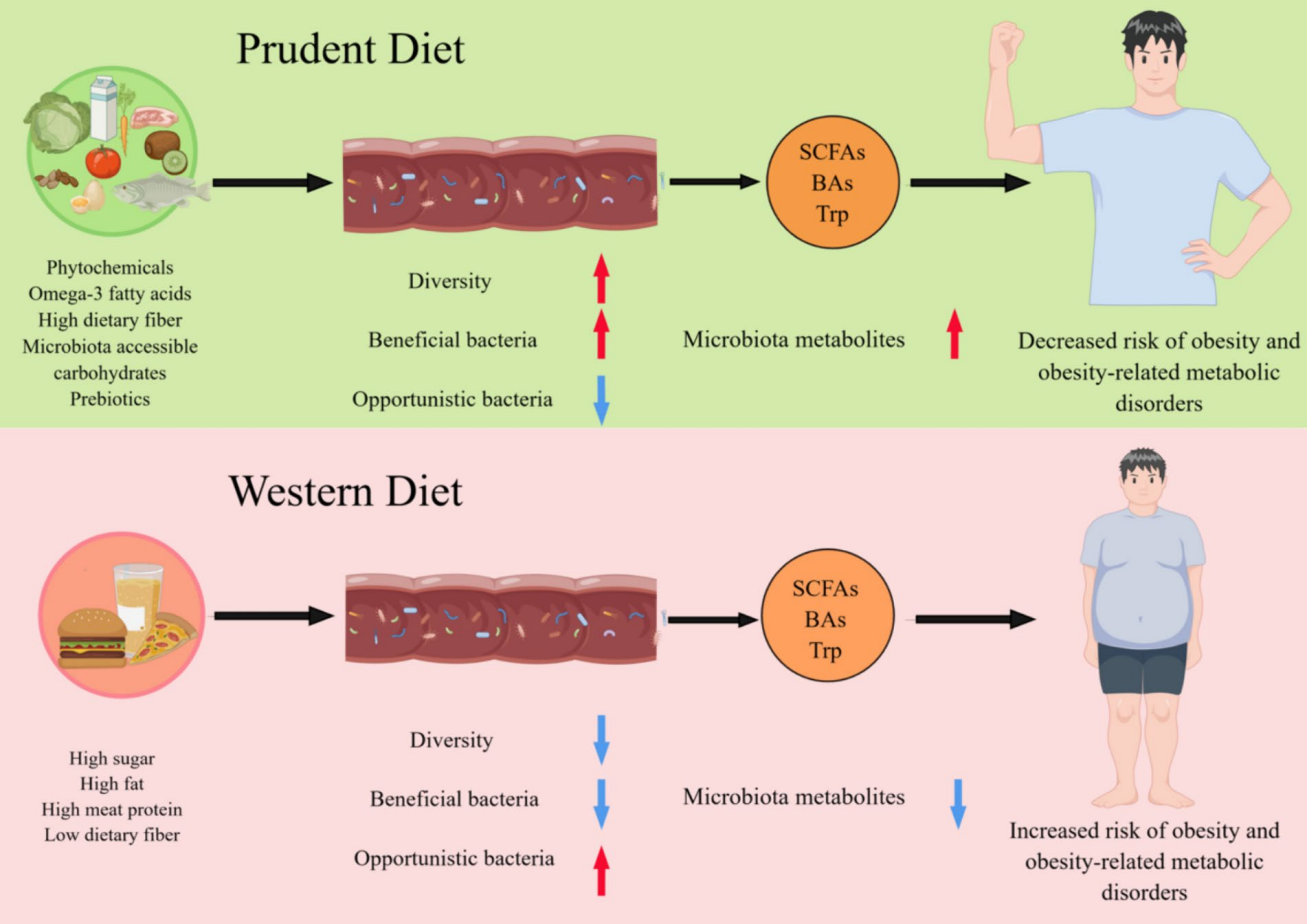
Different dietary patterns affect host health by reshaping the gut microbiome. A diet rich in phytochemicals, fiber, omega-3 fatty acids, microbiota accessible carbohydrates, and prebiotics promotes the maintenance of a healthy GM associated with increased diversity and functions, such as the production of short-chain fatty acids (SCFAs), Bile acids (BAs) and tryptophan (Trp). Conversely, the industrialization of the diet, with low fiber intake and high protein and sugar consumption, reduce the diversity of GM and alters their function is altered, resulting in a significant reduction in their ability to produce microbiota metabolites, which is associated with the development of obesity and related metabolic diseases.

Increasing evidence indicated that dietary patterns and habits have a significant impact on the diversity, composition, and richness of gut microbiota (GM). In a comprehensive review of the GM, it was found that the majority of GM are beneficial or commensal, playing essential roles in digestion, immune function, and metabolism (13). Opportunistic GM are typically present in low abundance and are kept in check by the beneficial microbes and the immune system (14). However, disruptions in the gut ecosystem, such as antibiotic use or dietary changes, can lead to an overgrowth of opportunistic GM, potentially causing health issues (15). The experimental model has shown that different GM communities develop after a period of intervention with various dietary patterns (16). Long-term dietary patterns, such as those that are high in protein, animal fats, carbohydrates or plant-based foods, significantly alter the GM (17). The GM contains trillions of microbes critical to host development and physiology. Evidence is growing that GM is an important environmental factor in human obesity since it may contribute to altering host energy harvesting and storage (18). The GM forms a complex web of relationships with each other and hosts, making it difficult to determine the causal role of GM in human obesity and associated metabolic disease. However, many studies have demonstrated that changes in the composition of the GM in animals occur after a shift in dietary patterns, suggesting that obesity may be prevented by interfering with the gut microbiota composition, which indicates a new potential effective treatment method (19) (Figure 1).

## 2 Changes of GM in obesity and related metabolic diseases

The content of *Bacteroides thetaiotaomicron*, *B. intestinalis* and *B. ovatus* in the intestine of patients with obesity are significantly lower than that of healthy individuals (20). *B. thetaiotaomicron* is one of the most abundant bacteria in the human gut and is a symbiotic bacterium that ferments glutamic acid (21). Studies have shown that gavage with *B. thetaiotaomicron* decreases plasma glutamate density and decreases diet-induced weight gain and obesity in mice (22). In addition, following bariatric surgical intervention, some of the obesity-related microbial and metabolic alterations in patients with obesity involve a decrease in the abundance of *B. thetaiotaomicron* and improved serum glutamate concentration (22). Transfer of the GM from obese mice into germ-free (GF) mice results in weight gain in GF mice, and the inverse is also true. After transferring fecal material from mice that have undergone Roux-en-Y gastric bypass into the gut of non-operated GF mice (23), the GF mice develop a weight loss and fat loss phenotype (24). These results support a causal relationship between gut microbes and specific metabolic outcomes (25).

Obesity is strongly associated with non-alcoholic fatty liver disease (NAFLD) (26). A comparison of the GM of NAFLD patients and healthy individuals has revealed several differences. At the phylum level, there is an increase in *Proteobacteria* in NAFLD patients (27). At the family level, NAFLD patients exhibit an increase in *Enterobacteriaceae* and a decrease in *Rikenellaceae* and *Ruminococcaceae*. At the genera level, NAFLD patients show an increase in *Escherichia*, *Dorea*, and *Peptoniphilus*, but a decrease in *Anaerosporobacter*, *Coprococcus*, *Eubacterium*, *Faecalibacterium*, and *Prevotella* (28). Experiments on rodents demonstrate a causal relationship between NAFLD and gut microbes. Breeding genetically modified mice to develop non-alcoholic steatohepatitis (NAFLD) with healthy wild-type mice results in liver steatosis and inflammation in healthy mice (29), indicating a shared GM between the two groups. These findings have also been confirmed by the fecal microbiota transplantation of these two groups of mice (30). After transplanting the GM from NASH patients into healthy GF mice, the healthy mice develop NASH symptoms, such as hepatic steatosis and inflammation, and these symptoms are exacerbated by a high-fat diet (31). Although mouse models cannot perfectly mimic the human gut environment, these studies confirm the impact of gut microbes on NAFLD.

Obesity is often associated with a high incidence of diabetes, characterized by hyperglycemia, relative insulin deficiency, and insulin resistance. Recent studies utilizing high-throughput sequencing on Chinese patients with T2DM have demonstrated that these patients have significantly reduced levels of *Roseburia intestinalis* and *Faecalibacterium prausnitzii*, *which* are involved in the production of butyric acid, but significantly increased levels of opportunistic pathogens such as *B. caccae*, various *Clostridiales*, *Escherichia coli*, and *sulfate-reducing Desulfovibrio* in the gut compared to healthy individuals (28). Moreover, the genetic differences between the GM of T2DM patients and healthy individuals are significant (>3%) (32).

Metformin is a widely used drug for the treatment of T2DM. Animal experiments have shown that high-fat diet (HFD)-fed mice given metformin have significant changes in blood sugar levels and GM compared to controls. Notably, oral administration of *Akkermansia muciniphila* to-fed mice achieve the similar effect as metformin (33). Clinical studies have also demonstrated that transplanting gut microbes from healthy individuals into patients with metabolic syndrome can increase insulin sensitivity and improve gut microbial composition (34, 35). These studies suggest that it is feasible to combat obesity and its associated complications by interfering with the GM to influence host metabolism.

## 3 Effects of dietary components on gut microbiota

### 3.1 Effect of animal-based diet on gut microbiome

Studies have shown that diet is a significant determinant of GM diversity and composition. Short-term or long-term dietary interventions lead to reshaping of the GM, which tends to recover its previous profile upon resumption of the prior eating habits (36). In a healthy individual, the majority of food is absorbed in the small intestine, and only about 10% reaches the colon as indigestible remnants, such as complex carbohydrates, protein residues, and secondary bile acids generated by hepatic lipid metabolism (37). These bioactive compounds strongly influence the GM’s composition, abundance, and activity, and thereby impact human health through the fermentation processes mediated by the GM (36).

Animal experiments have shown that mice fed a high-fat and high-sugar diet, or low-fat and high-sugar diet, exhibited significantly reduced levels of *Bacteroidetes* and increased levels of *Firmicutes* and *Mollicutes* (38). Other studies have found that the mice induced by a high-fat diet experienced a significant decrease in *Enterococcus* in the gut, while *Enterobacteriaceae*, *Escherichia*, *Klebsiella*, and *Shigella* levels increased significantly (39, 40).

An animal-based diet is characterized by high protein and fat intake. Excess protein in the colon can raise the pH of the intestinal environment, creating an alkaline environment that may facilitate the proliferation of pathogens and potentially affect the composition and metabolism of some GM (41). Consuming a lot of fat prompts the gallbladder to release bile acids into the duodenum, which are mostly reabsorbed in the distal ileum. The unabsorbed bile acids are metabolized by the GM into secondary bile acids (42). Taurine cholic acid, a primary bile acid, can provide a homing signal for gut microbiota and promote spore germination (43). In addition, primary bile acids can effectively control the growth of pathogenic gram-negative bacteria in the small intestine (44). Animal experiments have demonstrated that increased levels of cholic acid can significantly alter the community structure of the GM, leading to an increase in *Firmicutes* and a decrease in *Bacteroidetes* (45).

### 3.2 Effect of plant-based diet on gut microbiome

A plant-based diet is a dietary pattern in which seeds, fruits, or plant tissues are consumed either in their raw form or after being processed as the primary source of energy and nutrition for humans (46). Animal studies have demonstrated significant variations in the GM of mice fed a plant-based diet compared with those fed a control diet. When mice were switched from a control diet to a plant-based diet, there was a notable increase in *Bacteroides* and *Alloprevotella*, while *Porphyromonadaceae* and *Erysipelotrichaceae* showed a decrease (47). In a study examining the differences in GM between European children, who consume a Western style diet, and African children, who have a diet rich in fiber and resistant starch, the African children’s gut microbiome was found to be enriched in *Bacteroidetes*, *Genus Prevotella* and *Xylanibacter*, but lacking in *Firmicutes.* Additionally, the amount of short-chain fatty acids (SCFAs) in the fecal samples of African children is significantly higher than that of European children, particularly propionate and butyrate (48).

Recent studies have highlighted the potential of probiotic supplementation in modulating the GM to prevent obesity and improve metabolic function (49). Certain microorganisms, including *Lactobacillus*, *Bifidobacterium*, *Saccharomyces*, *Streptococcus*, and *Enterococcus*, have been found to be effective in limiting obesity development. These bacteria exhibit low antibiotic resistance and are non-pathogenic, rendering them suitable for the treatment of obese animals (50). For example, the oral administration of *P. pentosaceus* has demonstrated effective reduction in body weight gain and visceral fat content in mice subjected to a high-fat diet (51). Similarly, *Saccharomyces boulardii Biocodex* has been found to play a significant role in treating of obesity in T2DM mice (52). Notably, the amount of *Bacteriodetes* increases in the gut of mice treated with probiotic supplementation compared to control mice (53), while probiotic supplementation reduces the proportion of phyla related to obesity (*Firmicutes*, *Proteobacteria* and *Tenericutes*) (54). However, while some experiments have demonstrated the potential of probiotics in preventing obesity, the complex role of the relationship between GM and obesity requires further research (55).

Dietary fiber is commonly defined as having three or more monomeric units that are resistant to endogenous digestive enzymes and cannot be hydrolyzed and absorbed in the small intestine, according to most countries. Thus, most insoluble forms of dietary fiber cannot be digested by bacteria or digested slowly once they reach the colon, while soluble dietary fiber is used as a substrate by the GM to ferment metabolites such as SCFAs (18). A high intake of dietary fiber alters the living environment of the GM, leading to the expansion of microbial populations that can utilize these substrates. For instance, intervention with fructan and galactooligosaccharide has been found to increase the abundance of *Bifidobacterium-*spp. and *Lactobacillus* (56).

Inulin, a reserve polysaccharide found in plants, is a natural water-soluble dietary fiber that is resistant to hydrolysis or digestion by gastric acid but can be utilized by beneficial microorganisms in the colon, thereby improving the intestinal environment (57). Studies have demonstrated that inulin supplementation with propionate effectively improves insulin resistance in patients with obesity, reduces levels of the inflammatory marker interleukin 8, and is closely related to changes in the GM. Inulin also promotes changes in gut bacterial populations at the class level, increasesing *Actinobacteria* and decreases *Clostridia*, and at the order level, decreasing *Clostridiales* (58). Furthermore, inulin supplementation increases *Bifidobacteria* in the gut, effectively attenuates the increase in intestinal mucus permeability induced by a Western diet in mice and restores mucus growth (59).

Resistant starch is found naturally in foods, such as potatoes, bananas, rice and its positive effects on GM have been widely studied (60). Animal experiments have shown that rats fed a diet supplemented with resistant starch had an increase in *Bifidobacterium-*spp. Resistant starch from modified potatoes can increase the abundance of *Lactobacilli*, *Streptococci* and *Enterobacteriaceae* in the GM (61).

Polyphenols, as a phytochemical component, have been found to not only have anticancer effects, but also have positive effects on the GM. Studies have shown that long-term consumption of blueberry powder beverage can increased the abundance of *Bifidobacterium* and *Lactobacillus* in the gut, while drinking red wine had a similar effect (62, 63). Another study has proved that red wine polyphenols can significantly increase the abundance of *Bifidobacteria*, *Lactobacillus*, *Faecalibacterium prausnitzii* and *Roseburia* in the feces of patients with metabolic syndrome (64). The gut microbiota of patients with heart disease also changes after consuming red wine, and their redox homeostasis improves (65). However, the potential health benefits of polyphenols through modulation of the gut microbiota are limited and have constraints. For example, the substantial individual differences in gut microbiota and the numerous factors influencing gut microbiota make it challenging to isolate the effects of alcohol on metabolism (66). Therefore, further research is needed in the future.

Flavonoids, widely found in fruits, vegetables, legumes, nuts, and seeds, have been shown to play a positive role in regulating the gut microbiome. Studies have found that grape seeds are rich in proanthocyanidin extracts, which can significantly increase the abundance of *Bifidobacterium* and *Lactobacillus* in the gut (67). Another study found that participants who used extracts containing flavonoids and polyphenols for 3 weeks had lower levels of *Faecalibacterium*, *Odoribacter* and *Parvimonas* in their gut (68).

## 4 Dietary patterns interfere with gut microbiota to combat obesity and related metabolic diseases

Given that our dietary patterns continuously and indefinitely deliver a specific mixture of micro and macronutrient amounts to our gut ecosystem, it is useful to evaluate the impact of current dietary patterns on the GM associated with the host. Dietary patterns and eating habits have a significant impact on the diversity, composition, and richness of the GM, as described in detail in Table 1.

**Table 1:** Effects of dietary patterns on human health.

Dietary pattern	Gut microbiota	Metabolite	The effect on health
Fiber-rich diet	*Prevotella copri↑* *Eubacterium rectale↑*	SCFA↑ FA↑	Maintain gut microbial diversity and immune system function to prevent diet-induced obesity and related metabolic diseases (69)
Mediterranean diet	*Prevotella copri↑* *Faecalibacterium↑* *Bacteroides↑* *Roseburia↑*	Plasma cholesterol↓ High-density lipoprotein↓	Maintain the diversity and functionality of intestinal flora, reduce the host’s insulin sensitivity, blood sugar, and blood pressure levels, and prevent related metabolic diseases (70)
Ketogenic diet	*Bifidobacteria*↓	Circulating ketone bodies↑ Bifidobacterial↓ intestinal Pro-in-flammatory Th17 cells↓	It can effectively control seizures, reduce diet-induced weight gain and prolong the lifespan of experimental animals (71)
Every-other-day fasting diet	*Firmicutes↑*	Cetate ↑ Lactate↑ Monocarboxylate transporter 1↑	Activates the browning of white fat and improves metabolic homeostasis in mice (72)
Carbohydrate-restricted diet	*Streptococcus↑* *Lactococcus↑* *Clostridium↑* *Bifidobacterium↑*	DNL↓ β-oxidation↑	Reduces inflammation， Improves fat metabolism, balances oxidative stress in NAFLD patients (73)

### 4.1 Fiber-rich diet

Dietary fiber changes the intestinal ecological environment by providing substrates for the growth of microorganisms, and the microorganisms that use these substrates will better survive and grow in the intestines (74). Studies have shown that a fiber-rich diet helps maintain and increase the diversity of the GM, particularly some bacteria that produce SCFAs, which are linked to the health of the host. There is increasing evidence that industrialization of diets, low fiber intake, high protein intake, and high sugar consumption reduce the diversity of human GM and alter their functions, especially significantly reducing their ability to produce SCFAs, which leads to some chronic inflammatory diseases (69). In contrast, the production of SCFAs by the GM and high fiber intake improve mucus and antimicrobial peptide production as well as increase the expression of tight junction proteins. Furthermore, SCFAs reduce oxygen levels and maintain the functionality of the immune system (75).

When the diet shifts to a Western diet (low fiber and high protein), the ecological environment in the gut is disrupted, with consequent increased risk of infection, resulting in inflammatory bowel disease and impaired intestinal physiology (18). The consequences of a lack of fiber, known as microbiota accessible carbohydrates, have been studied by researchers who intervened in mice colonized with human microbiomes. A low-fiber diet leads to a significant reduction in microbial diversity, which does not return to previous levels when the mice are returned to a normal diet (76).

Microbial metabolism of dietary fiber produces biomolecules that are beneficial to host health, such as those found in grain bran, resulting in the release of ferulic acid (FA) from bacteria, such as *Lactobacillus fermentum* that contain the FA esterase gene (77). FA is a phenolic compound found in plant cell walls that enhances their rigidity and strength. In the digestive area, FA acts locally to adjust gut physiology, and it is transported in its free form into the bloodstream to control systemic condition. FA possesses antioxidant and anti-inflammatory properties, making it a potential therapeutic candidate for various chronic pathologies, including neurodegeneration, obesity, diabetes, and cancer. FA administration stimulates nerve cells in corticosterone-treated mice and prevents Ab-related toxicity in a model of Alzheimer’s disease (18). FA has shown anti-inflammatory properties in a model of ulcerative colitis as characterized by upregulation of Interleukin-10 and reduction of proinflammatory cytokines (78). Furthermore, studies have shown that FA prevents diet-induced obesity and has an antidiabetic effect as indicated by significantly improved serum insulin and glucose levels in diabetic rats treated with FA-related compounds (79). In conclusion, dietary fiber is a primitive compound that is important for protecting gut ecology, especially in modulating host physiology.

### 4.2 Mediterranean diet

The MD is a healthy, nutrient-rich dietary pattern that emphasizes the consumption of vegetables, fruits, fish, seafood, beans, nuts, and grains (80). In cooking, vegetable oils containing unsaturated fatty acids are preferred over animal oils containing saturated fatty acids, especially olive oil (81). Studies have shown that after MD intervention, human plasma cholesterol and high-density lipoprotein levels significantly decreases compared to the control group, and the MD regulates the composition and function of the GM. Researchers have calculated the co-abundance groups (CAGs) by analyzing the GM of the experimental group with 16S rRNA gene sequencing, demonstrating significantly lower levels of co-abundance group 2 (including *Prevotella* as the most abundant genus) in subjects who exhibit reduced homeostatic model assessment but significantly higher levels of co-abundance group 4 (including *Faecalibacterium*, *Roseburia*, *Bacteroides* and other *Clostridia*) at baseline in participants who exhibit reduced homeostatic model assessment and increased dietary treatment. In addition, variation in insulin resistance is linked to baseline levels of these microbial taxa (70). These researchers also suggested that MD intervention might help ameliorate insulin sensitivity in individuals with higher levels of several *Bacteroides* species, lower levels of several *Bacteroides* species and lower levels of *Prevotella* sp. and *P. copri.* The association of *P. copri* with insulin resistance has previously been reported (82), and this association has been recently demonstrated to be strain-dependent and correlated with the occurrence of genes involved in branched-chain amino acid biosynthesis (83). These findings demonstrate that the MD effectively improves the richness, composition, and function of the human GM to combat obesity and its associated metabolic diseases, which is of great significance to clinical practice in the era of personalized nutrition (84).

### 4.3 Carbohydrate-restricted diet

A carbohydrate-restricted diet is commonly used as an intervention for the prevention and treatment of NAFLD (73). Studies have shown that a short-term low-carbohydrate and high-protein intervention can induce changes in GM and significantly improve metabolism in obese NAFLD patients. Other approaches have also confirmed that short-term intervention with a low-carbohydrate diet promotes multiple metabolic benefits in NAFLD patients with obesity (85). New evidence shows that a low-carb diet improves metabolism in NAFLD patients by decreasing *de novo* lipogenesis and increasing β-oxidation. Notably, carbohydrate-restricted diets lead to an increase in folate-producing bacteria in the gut, which are absorbed by the gut and then increase circulating folate. This increase may be associated with subsequent improvements in lipid metabolism, oxidative stress balance and reduced inflammation (86).

### 4.4 Intermittent fasting

Intermittent fasting is a popular eating pattern for weight control that is effective and natural. There is substantial evidence that intermittent fasting optimizes energy metabolism and promotes health (87, 88). A previous mouse study demonstrated that every-other-day fasting intervention, significantly changes the GM of mice. This change results in an increase in two bacterial fermentation products, acetic acid and lactic acid, as well as an increase in monocarboxylate transporter 1 expression in white adipose tissue. Interestingly, GF mice are resistant to every-other-day fasting induction, and when the GM of every-other-day fasting-treated mice is transplanted into GF-free mice, the GF mice develop the same phenotype as before. Therefore, the dietary pattern of intermittent fasting provides a novel approach to promote brown adipose tissue browning and improve metabolism in humans by altering the GM (72).

### 4.5 Ketogenic diets

KDs are low-carbohydrate and high-fat dietary patterns that induce a dramatic shift in metabolic fuel utilization and increase circulating ketone bodies. Studies have shown that ketone bodies effectively inhibit the growth of *Bifidobacteria* in the gut and reduce levels of proinflammatory Th17 cells (71). Animals’ studies have also shown that KDs protect nerve cells by modulating the abundance of specific gut microbes that improve hippocampal γ-aminobutyric acid/glutamate levels (89). In addition to their use in controlling epilepsy, KDs have also been used for weight loss and other purposes. Animal experiments have demonstrated that KDs prolong lifespan and reduce the onset of metabolic diseases (90). All the evidence suggests that dietary interventions in the GM are promising for the treatment of obesity and its associated metabolic disorders.

## 5 GM metabolites

The GM performs many important functions, including participation in metabolic processes. The GM helps to biotransform many chemical compounds, as detailed in Table 2. They are also able to repurpose complex nutrients that humans cannot digest due to their metabolic capacity. For example, plant cell wall components such as cellulose, pectin, hemicellulose, lignin and mucins are repurposed into simple sugars that are then fermented to form SCFAs, such as acetate, propionate and butyrate (102). Additionally, the GM produces some neurotransmitters, including serotonin and gamma-aminobutyric acid (103). Microbes also stimulate enteroendocrine cells to produce key hormones, that are transported by body fluids and act on different organs over long distances (104). In this review, we will focus on three GM metabolites that are currently being studied: SCFAs, bile acids, and tryptophan.

**Table 2:** Effects of SCFAs on human health.

Type of SCFA	Effect on human health	References
Acetate	Prevents *E. coli* O157:H7 infection	(91)
	Involved in the synthesis of cholesterol	(92)
Butyrate	Provides the main source of energy for intestinal epithelial cells	(93)
	Mucin production and increases MUC2 gene expression	(94)
	Immune surveillance and immune regulation on tumor cells	(95)
	Suppresses the genotoxic activity of hydrogen peroxide and nitrosamines	(96)
	Has immunoregulatory effect	(97)
	Prevents and treats diseases, such as Crohn’s disease, distal ulcerative colitis Crohn’s disease and cancer	(98)
Formate	Associated with the production of methanogenesis, the concentration of which may be elevated during inflammation	(99)
Propionate	Reduces cholesterol synthesis in the liver	(100)
	Has anti-inflammatory and antibacterial properties	(101)
Valerate	Promotes the growth of intestinal epithelial cells and has a beneficial effect on the pathogenesis of diseases, such as cancer, colitis and cardio-metabolic diseases	(74)

### 5.1 Short-chain fatty acids

Most SCFAs are absorbed by the colon; with some used as an energy source for colonic mucosal epithelial cells and others entering the portal bloodstream (105). SCFAs affect appetite and energy intake via various mechanisms. Rodent experiments have shown that SCFAs stimulate the secretion of peptide YY and glucagon-like peptide 1. *In vitro* studies have further confirmed that SCFAs affect appetite and energy intake through the FFA3 (G protein-coupled receptor41) and FFA2 (G protein-coupled receptor43) (106). Furthermore, SCFAs stimulate the secretion of the adipose-tissue-derived satiety hormone, leptin, which has been confirmed in mouse, bovine and human adipocytes (107, 108) SCFAs also play an important role in regulating pH and increasing the uptake of calcium, iron and magnesium, and they are beneficial for glucose and protein metabolism in the liver. Furthermore, SCFAs affect the maintenance of the normal function, structure, and integrity of the intestines (102). Studies have shown that SCFAs production by the colonic microbiota modulates the activity of surrounding host enteroendocrine cells (109). Furthermore, SCFAs inhibit the development of pathogenic microorganisms, such as *E. coli*, Salmonella or Campylobacter, which compete for colonization sites in experiments simulating the growth of saprophytic flora (101). A previous study in mice has indicated that chronic oral butyrate administration prevents diet-induced obesity, progression of NAFLD and insulin resistance (110). Human *in vivo* studies have shown that acute rectal infusions of sodium acetate and SCFAs mixtures increase circulating concentrations of peptide YY in individuals who are overweight (111, 112).

In summary, SCFAs, as metabolites of intestinal bacteria, perform many important functions. Patients with obesity and obesity-related metabolic diseases commonly observe an imbalance in the GM, in which the number of SCFA-producing bacteria is significantly reduced. The composition and abundance of gut microbial populations, genetic factors, environmental factors, and host dietary habits can all affect the concentration of SCFAs.

### 5.2 Bile acids

The mammalian gut contains a large number of BAs, which can be converted by some GM into bioactive molecules that benefit the host (113). In mammalian intestines, some gut microbes metabolize primary BAs into secondary BAs, and both primary and secondary BAs are important bioactive molecules that regulate the host’s metabolic and immune responses (114). Differentiation and function of T cells, including inflammatory Th17 cells and anti-inflammatory regulatory T cells, are regulated by BAs. Th17 cells help prevent the invasion of extracellular pathogens, and Tregs help the host maintain immune tolerance (115). Secondary BAs, such as isoallolithocholic acid (isoalloLCA) and isodeoxycholic acid (isoDCA), modulate the differentiation of Tregs (116). In addition, 3-oxoLCA hinders Th17 cell distinction by blocking the function of the retinoic acid-related orphan receptor γt nuclear hormone receptor. 3-oxoLCA is absent from the caeca of GF C57BL/6 mice, indicating that gut bacteria may synthesize 3-oxoLCA (117). BAs can also be esterified by gut microbes, and esterified BAs are more hydrophobic; among which, ethyl-esters and long-chain fatty acid esters of LCA and polyesters of DCA account for approximately 25% of fecal BAs (118). Interestingly, gut microbes reduce the bactericidal effect of BAs by transforming DCA and LCA into isoDCA and isoLCA, respectively, (3b-OH epimers) via the iso-BA pathway (119). Research has shown that BAs damage bacterial membranes and alter intracellular macromolecular structures through detergent actions, representing the antibacterial effects of BAs. Therefore, only microbes that can tolerate high concentrations of BAs can survive in the gut. Free BAs are more disruptive to bacterial membranes, while taurine catabolism end products promote the proliferation of certain bacterial strains (120).

### 5.3 Tryptophan

Trp metabolites are among the most extensively studied GM metabolites. In the gut, gut microbes convert Trp into several biomolecules, particularly indole and its derivatives. Several indole derivatives are ligands for the aryl hydrocarbon receptor (AhR), including indole-3-acid-acetic, indole-3-acetaldehyde, indole-3-aldehyde, indole-3-propionic acid, and indole acrylic acid. Intestinal AhR activity is inseparable from microbial metabolism (121). Certain members of the human GM, such as *Clostridium sporogenes*, metabolize Trp to produce the tryptamine neurotransmitter (122). This bacterium also produces indole-3-acid-acetic and indole-3-propionic acid, two Trp metabolites that affect gut permeability and host immunity through oxidative and reductive pathways (123). Numerous studies have demonstrated that several indole derivatives produced by GM through Trp conversion contribute to the pathogenesis of metabolic syndrome (124).

Previous studies have demonstrated that indole itself stimulates enteroendocrine L cells to produce GLP-1, an incretin that stimulates pancreatic cells to secrete insulin. This mechanism involves the rapid inhibition of voltage-gated K+ channels stimulating GLP-1 secretion but is controlled by longer-term effects on ATP synthesis inhibition, thereby reducing GLP-1 secretion (125). Gut-derived 5-hydroxytryptamine affects host metabolism by inducing anorexia and satiety, independent of central effects (126). The levels of 5-hydroxytryptamine increase during fasting, and 5-hydroxytryptamine stimulates lipolysis in adipose tissue and gluconeogenesis in liver cells, contributing to glycemic control (127). In addition, human obesity is often accompanied by a decrease in peripheral 5-hydroxytryptamine, which reflects the important role of Trp in the pathogenesis of obesity. Some scholars have used mouse models to study the role of AhR in metabolic syndrome, but no clear conclusions have been reached, suggesting that the role of AhR on host metabolism is rather complex. Moreover, AhR is expressed in various cells involved in the pathogenesis of metabolic syndrome, such as enterocytes, hepatocytes, and immune cells (124).

## 6 Remaining questions and prospect

Dietary patterns play a crucial role in shaping the GM, influencing its richness and diversity. However, research on dietary patterns face several challenges. For example, when comparing the influence of dietary habits on GM of individuals from different regions, researchers must consider other factors, such as living conditions and the environment, besides differences in dietary habits. In addition, the animal models used in this review have limitations. The gut is a complex environment, and the composition of the GM, the host’s own immune and metabolic functions, and the host’s environment and lifestyle factors make it challenging for existing animal models to fully emulate the human condition (128). Studies of dietary patterns in animal models typically show more significant effects than clinical trials, and intervention doses relative to body weight are much lower than in clinical trials (129).

Due to the highly personalized nature of the human gut microbiome, the same intervention experiment may yield different results for different individuals (130). When a critical species is absent in an individual, a fixed dietary pattern may not produce the desired effect (131). Studies have shown that the composition of the GM is associated with insulin sensitivity in men but not in women (132). Another interesting finding was that after 12 weeks of intervention with a combined polyphenol supplement diet resulted in significant changes in the gut microbiota of men but not women (133). Research has shown that creating personalized diets based on eating habits, physical activity, and GM with the assistance of machine algorithms can effectively lower blood glucose responses (134). These studies highlight the importance of personalizing dietary patterns. Although the personalizing dietary patterns can be challenging, it has significant practical value. Developing personalized nutrition interventions targeting the gut microbiota is more effective in combating obesity and related metabolic diseases.

**Figure 2:**
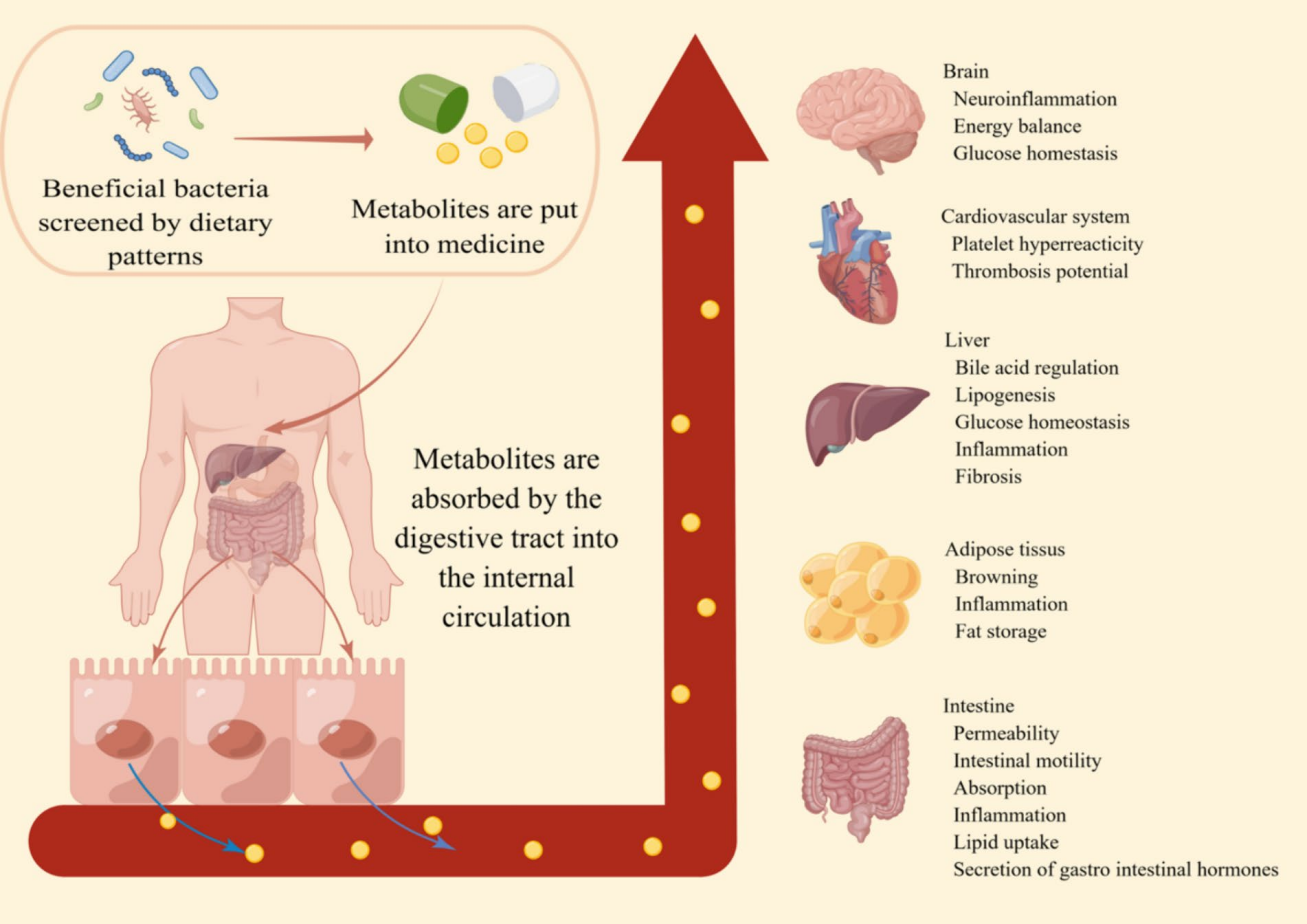
The prospect of dietary patterns against obesity. By incorporating the metabolites produced by beneficial bacteria screened through a healthy dietary pattern into pharmaceuticals, these small metabolic molecules are absorbed through the digestive tract into the systemic circulation. This enhances the body’s metabolic capacity, immune function, and various aspects of intestinal functionality. This makes the prevention and treatment of obesity and its related metabolic diseases simpler and more effective.

Another concern is that individuals with obesity and related metabolic diseases may find it difficult to maintain long-term dietary pattern changes. For example, long-term consumption of high doses of dietary fiber can lead to adverse side effects such as bloating, stomach pain, diarrhea, and constipation (135). Therefore, further studies are urgently needed to establish the relationship between dietary patterns and the gut microbiota, elucidate the role of the gut microbiota in metabolism, conduct a more comprehensive analysis of obesity and related metabolic diseases, and ultimately identify effective metabolic small molecules (Figure 2). Ultimately, these findings may be translated into personalized diagnostic and therapeutic tools to combat obesity and its associated metabolic disease.

## 7 Summarize

Despite the difficulties of studying the dietary patterns interfere with GM to combat obesity, increasing evidence suggests that dietary patterns interventions targeting the GM are a viable strategy to combat obesity and associated metabolic diseases. Methods such as the MD, KDs, are increasingly being utilized in clinical practice and have yielded impressive results. Additionally, research focused on elucidating the metabolic pathways through which the GM influences obesity and related metabolic disorders is rapidly advancing, leading to the identification of potentially powerful therapeutic targets. As our understanding of the interplay between diet and GM continues to deepen, it is possible that personalized nutritional approaches tailored to individual patients could be developed to combat obesity and related metabolic diseases more effectively.

## Author contributions

XLo: Conceptualization, Data curation, Formal analysis, Funding acquisition, Investigation, Methodology, Project administration, Resources, Software, Supervision, Validation, Visualization, Writing – original draft, Writing – review & editing. PL: Conceptualization, Data curation, Formal analysis, Resources, Writing – original draft. XLu: Data curation, Methodology, Supervision, Writing – original draft. ZL: Investigation, Methodology, Writing – original draft. XuL: Project administration, Resources, Writing – original draft. YL: Validation, Visualization, Writing – original draft. LG: Data curation, Methodology, Writing – original draft. WX: Investigation, Software, Writing – original draft. XiL: Conceptualization, Data curation, Formal analysis, Funding acquisition, Investigation, Methodology, Project administration, Resources, Software, Supervision, Validation, Visualization, Writing – original draft, Writing – review & editing.
